# Assessing performance in pre-season wrestling athletes using biomarkers

**DOI:** 10.11613/BM.2018.020706

**Published:** 2018-04-15

**Authors:** Ionas Papassotiriou, Artemissia-Phoebe Nifli

**Affiliations:** 1 School of Physical Education and Sport Science, National and Kapodistrian University of Athens, Athens, Greece; 2 Department of Nutrition & Dietetics, Technological Educational Institute of Thessaly, Karditsa, Greece; 3 Department of Biochemistry & Biotechnology, University of Thessaly, Larissa, Greece; 4 Biotechnology, Technological Research Center of Thessaly, Larissa, Greece

**Keywords:** exercise, glucose, lactic acid, creatinine, body composition

## Abstract

**Introduction:**

Although regular training introduces the desired changes in athletes’ metabolism towards optimal final performance, literature is rarely focusing on the metabolic responses off-competition. Therefore, the aim of this study was to evaluate biochemical indices during typical preseason training in wrestling athletes.

**Materials and methods:**

Twenty male freestyle and Greco-roman wrestlers (14 to 31 years) followed a typical session of the preparatory phase. Capillary blood glucose and lactate concentrations were assessed immediately before and after training. Protein, microalbumin, creatinine and their ratio were estimated the next day in the first morning urine.

**Results:**

Pre-training lactate concentrations were lower in Greco-roman than in freestyle wrestlers (1.8 (1.4 – 2.1) *vs.* 2.9 (2.1 – 3.1) mmol/L). Exertion resulted in a significant increase in lactate concentrations, by 3.2 (2.6 – 4.1) mmol/L in Greco-roman wrestlers and 4.5 (3.4 – 5.3) mmol/L in freestylers. These changes were found to correlate with athlete’s sport experience (r_s_ = 0.71, P < 0.001). Glucose concentrations were also significantly increased by 0.5 (0.1 – 0.8) mmol/L, in correlation with lactate change (r_s_ = 0.49, P = 0.003). Twelve subjects exhibited urine albumin concentrations at 30 mg/L, and thirteen creatinine concentrations around 17.7 mmol/L. The corresponding ratio was found abnormal in 4 cases, especially when creatinine excretion and body fat were low.

**Conclusions:**

Wrestling training is associated with mobilization of both lactic and alactic anaerobic energy systems. The regular comprehensive monitoring of biochemical markers would be advantageous in determining the efficiency of the preparatory phase and the long-term physiological adaptations towards the competition phase, or athlete’s overtraining.

## Introduction

During physical activity and exercise, energy systems collaborate to preserve energy supply to the muscle, while the intensity and duration of exercise define the degree of their contribution (*1*). A limited amount of readily available adenosine triphosphate (ATP) and creatine phosphate (PCr) cater muscles during fast twitches. Shortly afterwards, energy provision is achieved through the breakdown of circulating glucose and intramuscular glycogen. Long-term athletic training focuses on the development of motor and technical skills, though such adaptations include the optimization of energy resources through short-term training periodization and progressive cycles of preparation, competition and transition (*2*). Simultaneously, training periodization works as a safeguard against physical and mental fatigue, leading to injuries and chronic conditions.

Haematological and urine biomarkers have been proven valid for the quantification and monitoring of training load throughout a training period (*3*). Blood lactate is indicative of aerobic endurance and correlates strongly with heart rate variability, while lactate threshold improves throughout a season. Several hormones, measured in plasma or urine, predominantly norepinephrine, epinephrine, adrenocorticotropic hormone, testosterone, cortisol and corticosterone, and their ratios, reflect the presence of stressors in athletes life, though it is difficult to discriminate between actual physical or emotional burden (*2*, *3*). Whole blood or plasma creatinine and estimated glomerular filtration rate (eGFR) are particularly increased in strength sports, while creatine kinase (CK) may be elevated during acute changes in training load (*2*, *4*-*6*). However, because of the inter and intra-individual variations within the same or different seasons, the effect of exercise on biomarker concentrations, and the lack of reference values, little progress has been made towards the integration of these tools in sports practice and the discrimination among health, training, overtraining, and fatigue (*2*, *5*, *6*).

Since particular phases of training and certain sports are understudied, the aim of the current study was to provide novel evidence about biochemical indices during preseason and identify changes in two groups of Greco-roman and freestyle wrestlers for the first time. Wrestling is the oldest combat sport in the world and has high anaerobic energy requirements (*7*). There is no evidence on the biochemical responses during early training, while a few studies have focused on acute responses of the wrestling athlete, mostly lactate accumulation, during competition, sham matches or under strict laboratory conditions (*8*-*11*). It was hypothesized that a typical preparatory phase will affect the circulating blood lactate and glucose concentrations, and these changes would be detectable within the training time frame. It was further expected that these measures would be different between wrestling styles, as well as the magnitude of the exertion-induced shifts. Since during this mesocycle phase athletes are exposed to an increasing work load, the mobilization of creatine resources and the modification of renal function were anticipated. Previous sport experience would facilitate the transition towards the competition phase, thus resulting in moderate biochemical responses, while an increased body mass would have the opposite effect. For these purposes, minimally invasive techniques, that could be eventually applied in the field for athlete’s regular training monitoring, have been employed.

## Materials and methods

### Study design

The current study is an observational analytical cross-sectional study. The study protocol has been prepared according to the Declaration of Helsinki and approved by the Committee of Education & Research of TEI of Thessaly. Study purpose and procedures were communicated to individual sports clubs in Athens, Greece, and after initial approval volunteers were recruited. Athletes willing to participate provided us with written informed consent. In case of underage individuals, additional consent from parents (or guardians) was obtained.

By the end of the summer transition period and upon the beginning of conditioning, participating athletes have been treated individually, in order to retrieve personal information, anthropometrics, compliance with the training schedule, and dietary habits. In agreement with the coaches, preliminary biochemical indices and exercise intensity were estimated in a random sample of athletes during the first month of the preparatory phase in September 2015 (general preparatory phase). Finally, each group of athletes of either style has been screened at week #7 of the preparatory phase (October 2015), designated also as week #3 of the specific preparatory phase, during a mid-week preparatory routine, as described below. One week later, athletes followed their own personal bi-cycle or tri-cycle plan. Interviews, measurements and sampling took place in sport clubs’ premises.

### Subjects

Twenty semi-professional male wrestlers of competitive level in total, eleven Greco-roman wrestlers and nine freestylers (from two different clubs), aged 17 (14 – 31) years, participated in this study. Criteria for inclusion were a) male sex b) the absence of a medical condition or seasonal infectious disease, as confirmed by team physicians and c) the participation in a national wrestling competition in the specified style, free or Greco-roman. Some wrestlers participated in one competition, others in more than one. Occasionally, a wrestler may have failed to qualify for the finals in one competition, but he was successful in the next one, a few months later. Athletes that failed to be admitted to the finals at least once during the previous 12 months in the specified wrestling style have been excluded. In order to achieve a uniform preparatory phase timeline, as per coach’s suggestion, athletes missing two or more appointments per week have been excluded, as well as those who have not complied with the training schedule during the last two years. For the same reason, successful athletes with ongoing multi-peaking plans (competing several times per year) have been also excluded. Due to the above elimination criteria, only 20 individuals within a wide age range have been cleared to participate in the study. No athlete was using medication, either prescribed or over-the-counter. Diet and supplementation was monitored by athletic trainers and team physicians. During the study, creatine supplementation was never prescribed or self-reported, while 5 athletes consumed electrolyte supplements, of which 2 also consumed a protein supplement. Age and experience were not applied as exclusion criteria, in order to assess their potential effect on biochemical parameters.

### Anthropometry

Anthropometric measurements were taken in separate appointments for each athlete at morning hours, before food consumption and after evacuation. Height and weight were measured with a digital beam scale (WB-3000; Tanita Europe B.V., Amsterdam-Zuidoost, The Netherlands). Four skinfolds’ (biceps, triceps, subscapular, suprailiac) thickness was measured with a Lange skinfold caliper (Beta Technology, Santa Cruz, USA), and subsequently body fat percentage was estimated using the Durnin-Womersley equation:
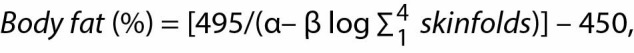
where *α* and *β* represent the suggested age constants (males aged < 17 years: *α* = 1.1533, *β* = 0.0643; males 17 - 19 years: *α* = 1.1620, *β* = 0.0630; males 20 - 29 years: *α* = 1.1631, *β* = 0.0632; males 30 - 39 years: *α* = 1.1422, *β* = 0.0544). Classification according to body fat was performed using separate standard curves for adolescents and adults (*12*, *13*). In addition, due to the inclusion of underage athletes in the study, the classification of the sample was based on the corresponding body mass index-for-age z-score (BAZ) values, as described by the World Health Organization (WHO), instead of mere body mass index (BMI) (*14*).

### Exercise intensity

Exercise intensity was determined with an armband accelerometer (SenseWear^TM^; BodyMedia Inc., Pittsburgh, USA), to estimate training metabolic equivalents of task (METs) (*15*). For the given data, intensity was recorded in a moderately experienced athlete. Heart rate monitors could not be worn, because of the nature of the sport and the close contact with the opponent.

### Training

Both freestyle and Greco-roman wrestling training workouts included 10 minutes (min) jogging, 20 min technical warm up exercises, 30 min skill exercises, two 3 min matches and about 5 min dynamic and static stretching exercises. The weekly microcycle comprised 5 training and 2 rest days. Reported participation ranged from 3 - 5 times *per* week. Athletes joined training practice two hours after the consumption of a meal or snack. The athletes did not consume beverages containing carbohydrates since the last meal/snack and until the completion of training, while they had free access to water during practice intervals.

### Estimation of glucose and lactate concentration in capillary blood samples

Blood glucose and lactate measurements took place immediately before and after a typical afternoon training session, scheduled between 18:30 and 20:00. Capillary blood was collected through middle finger prick using disposable lancets (Accu-Chek^®^Safe-T-Pro Uno; Roche Diagnostics GmbH, Mannheim, Germany) according to Clinical and Laboratory Standards Institute and WHO guidelines. Capillary glucose was estimated using a portable enzymatic meter (Contour^TM^XT, Ascensia Diabetes Care Holdings AG, Athens, Greece), after being tested against a set of control solutions (high concentration solution with expected range 6.8 - 8.6 mmol/L and low concentration solution with expected range 4.7 - 6.4 mmol/L), and the corresponding strips Contour^®^next (Lot No: DP4HFED03A; Ascensia Diabetes Care Holdings AG, Athens, Greece) in the first blood drop, as recommended by the manufacturer (*16*). After wiping the skin with a clean gauze, a second blood drop was applied directly to BM-Lactate strips (Lot No: 201 323-01; Roche Diagnostics GmbH, Mannheim, Germany) and lactate was estimated using the Accutrend^®^Plus portable enzymatic meter (Roche Diagnostics GmbH, Mannheim, Germany), calibrated daily with the internal control strip, included in each individual strip package (*17*).

### Estimation of creatine metabolism and renal clearance

Nineteen out of the twenty wrestlers provided a first morning urine sample the day after the typical training session. Morning urine collection provides comparable results to a 24 h collection sample, and ensures appropriate sampling and storage by the participants (*18*). Urine was collected in sterile urine cups (Alfa-Gauze, Karabinis Medical S.A., Paiania, Greece), refrigerated at 4 ^o^C when transferred and analysed the same day, 30 min after sample reached room temperature. Creatinine and albumin concentrations were assessed using semi-quantitative paper chromatography (Microalbustix^®^, Lot No: 408031; Siemens Healthcare Diagnostics Inc., Tarrytown, USA), according to manufacturer’s instructions (*19*, *20*). The test detects albumin in concentrations from 10 to 150 mg/L and creatinine from 0.9 to 26.5 mmol/L, across a given scale of categorical anchors. The assay was performed after confirming that protein concentration was below 300 mg/L using the standard dipstick method (Multistix®10 SG, Lot No: 404015; Siemens Healthcare Diagnostics Inc., Tarrytown, USA). A qUantify bi-level control (975X; Bio-Rad Laboratories Inc., Hercules, USA) was used to monitor dipsticks condition and precision.

### Statistical analysis

Statistical analysis was performed using IBM^®^ SPSS Statistics^®^ for Windows, Version 22.0. (IBM Corp., Armonk, NY). Because of the low number of participants, summary data are presented as median and interquartile range (Q1–Q3), and nonparametric tests have been applied. Mann-Witney U test was performed to detect differences between variables of different sport styles. Wilcoxon signed-rank test was performed to reveal differences between paired samples of variables, either within the whole sample, or in style subgroups. Spearman rank correlation was applied to test the association between two variables. A P value < 0.05 was considered statistically significant.

## Results

### Anthropometrics and training intensity

Anthropometric characteristics of the participating athletes are displayed in Table 1, for each wrestling style and in total. Variables did not differ across sport style categories. Calculated BAZ ranged from a minimum of - 0.87 to a maximum of 2.80. According to these values, the weight for-age z-score and the height for-age z-score, no adolescent was underdeveloped, underweight, or underfat. Based on BAZ, 9 of the participants had normal weight, 5 were categorized as overweight and 6 were obese. According to body fat percentage, 12 wrestlers were normal and 8 overfat. Metabolic equivalents of task were estimated at 8.20 for the freestyle wrestlers’ routine and 7.00 for Greco-roman wrestlers’ conditioning. According to Ainsworth criteria, as adopted later by the American College of Sports Medicine, the participating wrestlers followed a vigorous training schedule, independently of the style (*21*).

**Table 1:** Basic anthropometric characteristics of athletes

	**N**	**Age (years)**	**Weight (kg)**	**Heigth (cm)**	**BMI (kg/m^2^)**	**BAZ**	**Body fat (%)**
**Freestyle** **wrestlers**	9/20	17 (15 - 28)	77.8 (71.9 - 83.0)	179 (170 - 183)	25.90 (22.40 - 27.10)	1.12 (0.46 - 1.45)	16.80 (14.20 - 18.50)
**Greco-roman wrestlers**	11/20	15 (14 - 31)	73.2 (66.2 - 85.7)	172 (166 - 174)	25.90 (24.05 - 28.30)	1.79 (0.78 - 2.18)	23.00 (18.65 - 26.30)
**Total**	20/20	17 (14 - 31)	75.1 (69.6 - 85.1)	173 (167 - 179)	25.90 (23.45 - 28.25)	1.13 (0.63 - 2.17)	18.90 (15.33 - 25.00)
**Mann-Whitney U**	-	24.0	39.5	34.5	48.0	43.0	26.5
**P**	-	0.056	0.456	0.261	0.909	0.656	0.080
Age is presented as median (range). Data are represented as median (interquartile range). BMI - body mass index. BAZ - body mass index for age. P < 0.05 was considered statistically significant.							

### Lactate production, clearance and adaptations

**Figure 1:**
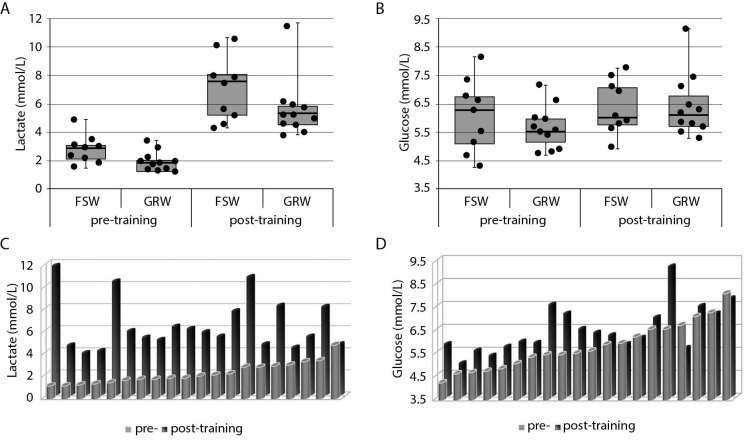
(A) Estimation of lactate and (B) glucose concentrations before and after a freestyle (FSW) or Greco-roman (GSW) pre-season wrestling training session. Box plots include median (IQR) and whiskers show the lowest and highest values per group sample. Individual fluctuations of (C) lactate and (D) glucose, as a result of training. Front raw bars in (C) and (D) represent basal values arranged in ascending order.

Absolute capillary lactate and glucose concentrations, before and after the completion of a training session are displayed in Figure 1. In the whole sample, lactate concentrations were 2.0 (1.7 – 2.9) mmol/L before training and 5.5 (4.6 – 7.7) mmol/L after training. A Wilcoxon signed-rank test showed that exertion resulted in significantly higher lactate concentrations (Z = - 3.88, P < 0.001), and mean lactate rank was 1.00 and 11.00 before and after training, respectively. Similarly, significantly higher lactate concentrations have been detected at the end of practice in both freestyle (Z = - 2.55, P = 0.011, rank_pre_ = 1.00 and rank_post_ = 5.50) and Greco-roman wrestlers (Z = - 2.94, P = 0.003, rank_pre_ = 0.00 and rank_post_ = 6.00). The greatest variability was observed in freestyle wrestlers, especially after workout. When styles were compared, baseline lactate concentrations were different across categories at the baseline (U = 21.00, P = 0.031), but not at the end of practice (U = 31.50, P = 0.175). No association between pre- and post-training lactate concentrations was found (r_s_ = 0.10, P = 0.675).

Glucose concentrations prior to training were found normal, as compared to random postprandial standards, starting from 4.3 mmol/L, for the athlete with the highest pre-training lactate concentrations, whereas one subject could raise concerns for a pre-diabetic state (Figure 1B). After routine completion, lower glucose concentrations were detected in one quarter of the participants (Figure 1D), the greater down by 1.2 mmol/L. The rest of the athletes had higher glucose concentrations. In the whole sample, Wilcoxon signed-rank test showed that training routine produced a statistically significant change in circulating glucose (Z = - 2.75, P = 0.006, rank_pre_ = 6.30 and rank_post_ = 11.90). This effect was significant in Greco-roman wrestlers (Z = -2.80, P = 0.005, rank_pre_ = 1.50 and rank_post_ = 6.45), but not in freestylers (Z = - 0.89, P = 0.374, rank_pre_ = 3.75 and rank_post_ = 6.00). No significant differences have been revealed when capillary glucose concentration of different styles was compared (U = 41.00, P = 0.552 and U = 47.00, P = 0.882, before and after training, respectively).

**Figure 2:**
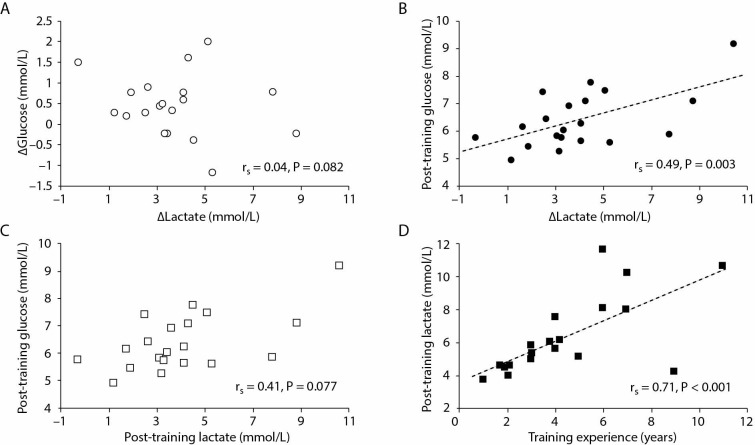
(A) Individual lactate changes due to training plotted against the corresponding glucose concentration shifts. No correlation was identified, P > 0.05. (B) Individual post-training glucose concentration plotted against lactate change. A positive correlation was found (P < 0.05). (C) Individual glucose and blood lactate concentrations, after the completion of training. No correlation was found (P > 0.05). (D) Post-training lactate concentrations as a function of experience. There was a positive correlation (P < 0.05).

With one exception, participants responded to training overcoming the clinically relevant lactate threshold of 4.0 mmol/L and the maximal raise of glucose concentrations by 2.6 mmol/L coincided with the maximal raise in lactate by 10.5 mmol/L. However, net lactate increase could be observed with either elevated or diminished glucose concentrations and no correlation between lactate difference (*Δ*lactate) and glucose difference (*Δ*glucose) was observed (Figure 2A). A moderate positive correlation was found between *Δ*lactate and circulating glucose at the end of the training (Figure 2B). Final glucose concentrations were not found to associate with absolute post-training lactate concentrations (Figure 2C). In addition, a good correlation between post-training lactate concentrations and experience in the sport was found (r_s_ = 0.71, P < 0.001, Figure 2D).

### Creatine mobilization and renal clearance

**Figure 3:**
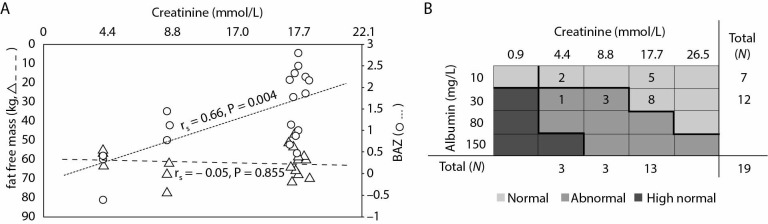
(A) Morning spot urine creatinine concentrations plotted against lean body mass (left vertical axis) or BAZ (right vertical axis). Creatinine has been estimated using a semi-quantitative assay, able to detect 0.9 to 26.5 mmol/L of the metabolite, across a scale of 0.9, 4.4, 8.8, 17.7 and 26.5 mmol/L anchors. Only body size was found to correlate significantly with PCr mobilization, P < 0.05. (B) Estimation of albumin to creatinine ratio in morning spot urine. A ratio < 3.39 mg/mmol is considered “normal”, a 3.39 - 33.94 mg/mmol ratio indicates microalbuminuria (“abnormal”), while a ratio ≥ 33.94 mg/mmol indicates clinical albuminuria (“high abnormal”).

Thirteen athletes showed elevated urine creatinine concentrations, around 17.7 mmol/L (Figure 3). No athlete with extreme creatinine concentrations (> 26.5 mmol/L) was found, while lower creatinine concentration, as expected in the general healthy population, was measured in 6 participants. After analysis of somatometric characteristics, a good positive correlation between BAZ and creatinine was detected (r_s_ = 0.66, P = 0.004; Figure 3A). No evidence of proteinuria was found, when urine protein was assessed using the standard dipstick method (Multistix^®^10 SG). However, when the relevant assay for microprotein concentrations was applied, a leakage of albumin, approximately 30 mg/L, was shown in 12 athletes. Considering the corresponding creatinine values, the calculated albumin to creatinine ratio (ACR) was ≥ 3.39 mg/mmol in 4 of the cases, and classified within the abnormal range. No wrestler exhibited highly abnormal ACR.

## Discussion

In wrestling athletes of competitive level, a typical training session results in the increase of blood lactate. This response is indicative of the importance of anaerobic glucose metabolism in this sport, either freestyle or Greco-roman, and correlates with athlete’s previous experience. In addition, the magnitude of the lactic response has an impact on glucose metabolism, resulting in the significant increase of circulating glucose at the end of practice. Elevated creatinine excretion is persistent in the morning urine of 70% of the athletes the day after the training, and associated with athlete’s body size, rather than muscularity, while athletes with lower creatinine concentrations are likely to exhibit abnormal ACR. Such transient changes in wrestler’s metabolism may present with physiological consequences. The observed microalbuminuria and elevated blood glucose concentrations imply renal hyperfiltration. However, further studies would confirm their potential involvement in the long-term adaptations to exercise and the need for timely and effective monitoring.

Pre-training lactate values in Greco-roman wrestlers are closer to the ones reported in the literature, while freestylers appear with significantly higher values, as shown once before (*9*-*11*). In this study, all athletes have been advised to minimize load and stay calm before the initial measurements, while they report no injury or disease and are able to exercise at high intensity levels. A diurnal effect between styles is excluded, as all basal blood values have been collected during afternoon practice between 18:30 and 20:00. A more comprehensive approach, including individual power output history, and the training experience, its frequency and strength, would facilitate the interpretation of baseline values and their integration in sports practice. Such approach would be also useful in clinical practice for the re-production of normative values, as when applying the clinically relevant cut-off of 2.3 mmol/L, 6 out of 9 freestyle wrestlers, but only 2 out of 11 Greco-roman wrestlers, should be classified as “hypoxic/ischemic” (*22*).

Freestyle wrestlers exhibit higher median post-training lactate concentrations than Greco-roman ones, about 2.3 mmol/L, but not significantly different. Following a shorter training protocol, a 20 minute warm up (10 minute light dynamic warm up and stretching exercises and 10 minute special wrestling warm up) and three 2 minute wrestling matches, Ghorbani *et al.* reported a mean lactate of 15.1 mmol/L immediately after the session in freestyle wrestlers (*11*). These values are higher than the ones recorded in our case (5.3 (4.6 - 5.9) mmol/L), whereas no athlete exceeds 10.7 mmol/L. Similarly, another study examining the performance of Greco-roman wrestlers showed a wider change in lactate concentrations, 5 min after a series of matches, up to 9.2 mmol/L, depending on the weight category (*10*). It is possible that in the subjects participating in this study, lactate clearance, although reduced, counterbalances lactate production during the 75 min typical training protocol. It is important to note that final lactate concentrations have been obtained at the end of the practice, after stretching session, and not immediately after the sparing matches. On the other hand, it is also possible that the extended regular routine affects athlete’s stamina and the ability to perform an all-out fight. Experienced athletes are more likely to respond with extreme lactate concentrations and this association is confirmed during the standard practice as a good positive correlation between post-training lactate and years of experience (*8*).

Lactate increase presumes the mobilization of glucose supplies, either from periphery, or within intramuscular glycogen stores (*1*). Shortage of these reservoirs could further enhance liver glycogenolysis and gluconeogenesis, while in parallel lactate should be converted back to glucose. Since the wrestlers participating in this study have not consumed any food or beverage, thus readily available carbohydrates or precursors, it may be concluded that the observed changes in blood glucose originate exclusively from the aforementioned intrinsic depots and mechanisms. Singular responses show high variance (Figure 1D), but pair analysis reveals statistical significance. In a study of 60 wrestlers, Karnincic *et al.* reported baseline glucose at 5.3, 5.5 and 5.6 mmol/L for lightweight, middleweight and heavyweight wrestlers respectively; while the same values shifted to 8.8, 8.3 and 8.7 mmol/L 5 min after matches (*10*). In agreement with these observations, our data reveal also a positive correlation between post-training glucose concentrations and change of lactate concentration (P = 0.003, Figure 2B), but not with absolute lactate values after training (P = 0.077, Figure 2C), and the greatest shift in lactate by 10.5 mmol/L coincides with the maximal glucose elevation by 2.7 mmol/L, at 9.2 mmol/L. In addition, the individual with the highest lactate concentrations (4.9 mmol/L) and the lowest glucose (4.3 mmol/L) before training responds with a 1.5 mmol/L glucose increment and a small lactate diminution. When these values are transformed according to the linear trapezoidal model, the positive association is not statistically significant (r_s_ = 0.29, P = 0.214). A more frequent sampling would be helpful in future investigations of both metabolite kinetics. Understanding the contribution of lactate to gluconeogenesis during practice would be also helpful to define the needs of wrestling athletes in carbohydrates and the appropriate time for supplementation.

In addition to lactate mobilization, wrestlers appear to depend on the ATP-PCr energy system during training, as evaluated the day after, through increased creatinine turnover in morning urine. This effect appears activity-specific, as in a previous study creatinine concentrations were below 4.4 mmol/L in cyclists, basketball players, and grappling wrestlers (*23*). Higher creatinine clearance may result from diet and mild physical activity and seems to be spontaneously resolved (*24*). Protein consumption or supplementation is not associated with urine creatinine concentrations in this sample. On the contrary, a significant good positive correlation between urine creatinine concentrations and BAZ is detected, the opposite for fat free mass index, while no correlation with lean mass is found (Figure 3A). Besides the large amount of data accentuating muscle mass as a predominant factor in serum creatinine modulation, our data designate the effect of body size, and subsequently the size related effort, as previously shown in elite athletes (*4*).

Exercise may also modify renal clearance. In normal subjects, protein leakage has been detected soon after exhaustive exercise (*25*). More specifically, 40% of the subjects have been tested positive for microalbuminuria, albuminuria was also observed, however ACR normalized completely 24 hours later. In our study, albumin concentration is found around 30 mg/L in 12 athletes (Figure 3B), and it could be regarded *per se* as borderline. After subtraction with creatinine concentration, ACR falls within the “normal” range for eight of the Greco-roman and freestyle wrestlers. Athletes with “abnormal” ACR show creatinine below the first decile, although creatinine values tend to accumulate around 17.7 mmol/L, within the upper quantiles of the general white population distribution (*26*). In a previous work similar low creatinine values have been detected in “normal” young athletes with no protein leakage (*23*). However, it has been reported that this standard correction with creatinine, applied to discriminate between prodromal and ongoing renal conditions, may also mask the effect of vigorous exercise on protein excretion (*27*). If this is true, and considering the magnitude of documented creatinine concentrations herein, the prevalence of microalbuminuria would be higher. Further studies will be needed to elucidate the impact of training routine on the time course of protein clearance and the possible implication of body size and composition.

Evidence about the fluctuations of biomarkers in response to training load and phase are of particular importance for their productive integration in sports practice (*2*). The current work provides new insights on the metabolism of wrestling athletes of competitive level, however it presents with some limitations. The total sample size could be considered adequate, though the size of wrestling subgroups could raise concerns regarding the power of statistical analysis. Because of the nature of the sport, similar studies in the field usually include low number of participants (*9*, *28*). Studies that include a higher number of athletes are rare and comprise international athletes with diverse background (*8*). If athletes with a multi-peaking schedule were screened, a seasonal effect would interfere with the collective interpretation of the data. The same would apply to volunteers from different associations. Larger studies focus on the physical parameters of the athletes, rather than biochemical indices (*29*). Thus, considering that athletes should follow the same routine for a long period, and compete within the same age and weight category during the final matches, the current sample size is one of the highest that could be achieved in Greece.

Regarding the diagnostic techniques and procedures employed herein, glucose and lactate have been estimated in capillary blood using point-of-care tests (POCT), while creatinine clearance, microalbuminuria and proteinuria have been tested in morning urine using a semi-quantitative method. These methods are considered less accurate than the respective analytical laboratory tests in plasma and urine, and may occasionally lead to over- or underestimation of the measures for analytical or pre-analytical reasons. However, they are less invasive, and also time and cost effective. Appropriate instrument maintenance, calibration, and sampling by trained individuals eliminate the occurrence of diagnostic errors and justify their reliability in the field (*16*, *17*, *19*, *20*). Besides the practicality of POCT, all administrative partners (university, guardians, and club managers) considered the interference of a phlebotomist critical for athletes’ stress levels; the effect of the white coat on hypertension is well established, while there have been concerns about the effect of the white coat on glucose concentrations (*30*). Furthermore, despite the initial plan to collect 24-hours and morning urine samples to assess protein, albumin, and creatinine concentrations, adverse participants’ compliance to the protocol led to opt finally for a single morning sample. Therefore, it may be also concluded that the selected experimental procedures reduced sampling bias.

The data presented in this study were collected under real training conditions. Thus, they reflect the exercise-induced metabolic responses during the preparatory phase and could be of practical use in clinical laboratory and sports science routine. They show that a typical wrestling training session leads to the increase of blood lactate concentration above the lactate threshold and further promotes gluconeogenesis. As experienced individuals are more prone to lactate shifts, lactate accumulation could be employed in the field for the tailored monitoring of wrestler’s effort and progress. The occasional elevation of resting lactate concentrations further supports the need of regular and comprehensive monitoring to assess overtraining or fatigue. Moreover, there is evidence that exercise may modulate protein clearance; however creatinine excretion could mask this effect, especially in sizable athletes. Perspective studies will determine whether these findings are interrelated and under hormonal control, as well as the validity of normative values for their interpretation in the exercising population.
